# Botswana children's needs: orphans and vulnerable children dominated child welfare system

**DOI:** 10.1016/j.heliyon.2020.e03801

**Published:** 2020-04-22

**Authors:** Nankie M. Ramabu

**Affiliations:** Leeds Beckett University, City Campus Leeds LS1 3HE, United Kingdom

**Keywords:** Health sciences, Social science, Botswana, Child welfare system, Children's needs, General child population, OVC

## Abstract

Children's needs in Botswana received considerable attention in the past three decades owing to the HIV/AIDS epidemic in the country. These decades of legal and policy practice processes focused on orphan and vulnerable children (OVC), ensured that the needs of the general population of Botswana children are grossly understudied, underestimated, and therefore, remain unaddressed. This study sought to determine the needs of the general population of children. Fifty-two visual arts, six semi-structured interviews, twenty-six in-depth interviews and two focus group discussions were conducted in two purposively selected sites with children, policy-makers, practitioners, community leaders and caregivers. The data were analyzed using deductive content analysis approach. Children expressed the need for basic, safety, love and belonging needs. Given some pockets of poverty that exists in Botswana, it is likely that children in the general population have needs similar to those of OVC. Therefore, child welfare program should also target children who are not considered OVC.

## 1 Introduction

Globally, child welfare systems emerged with the intention of taking care of children's psychosocial needs. In the global north, the welfare systems evolved over a long period of time (Schewchuk, 2014). These systems are guided by international instruments especially the United Nations Convention on the rights of a child (UNCRC). However, in the global south, Botswana included, the child welfare systems came into existence after independence which makes them fairly new and behind the global north child welfare systems. For instance, Botswana formal child welfare system has been documented from the late 1970s (Mogobe & Tshiamo, 2006 After Botswana's independence in 1966, child welfare was not for vulnerable children but all child population.

The system was universally welfare-based with focus on addressing children's basic needs, education, health needs such as immunizations from childhood illnesses, growth monitoring and promotion (Mogobe & Tshiamo, 2006; Nthomang, 2007). Therefore, the protection offered to children was food-aid and humanitarian responses to problems of food insecurity (Nino-Zarazua, 2012). In addition, under the leadership of the African Union and Southern African Development Community (SADC), Botswana designed and implemented the OVC and Youth programs targeting first, AIDS orphans and then marginalized child population (Botswana, 2008; SADC, 2011). Thus, Botswana Child Welfare System was expounded with the escalating HIV/AIDS OVC (Botswana National Plan of Action for OVC, 2010–2016)... According to the Botswana government, an orphan is a “*child below 18 years who has lost one (single) or both parents (married couples), these parents are either biological or adoptive*” (Botswana, 2008; Botswana, 2010). Whereas the country defines a vulnerable child as “*a child below age of 18 years who; lives in an abusive environment; lives in a poverty-stricken family and cannot access basic services; heads a household; lives with a sick parent(s)/guardian; is infected with HIV and lives outside family care*" (Botswana, 2008).

These OVC children's needs which are not mean tested (Mupudsizwa & Ntseane, 2013) include monthly food basket, clothing, toiletry and support with educational needs and lately psychological support (Ministry of Local Government, 2008a, Ministry of Local Government, 2008b). These OVC children are primarily cared for within the existing extended family structure, which shows the importance Botswana society places on the kinship structure. This OVC program is regulated by successive National Plan of Action for Orphans (Botswana, 2008; Botswana, 2010). In addition, Botswana operates a successful school feeding program for all government schools in the country where primary and secondary school children are provided with one meal a day (Botswana, 2012).

As a result of these efforts by Botswana government to provide for basic needs of children, the country has been rated child-friendly, with a child-friendly index score of 0.635 in 2008; 0.6881 in 2013 and 0.6176 in 2018 (http://www.africanchildinfo.net/). However, the pockets that exist across the country have implications for child welfare.

### 1.1 Children's needs

**Figure 1:**
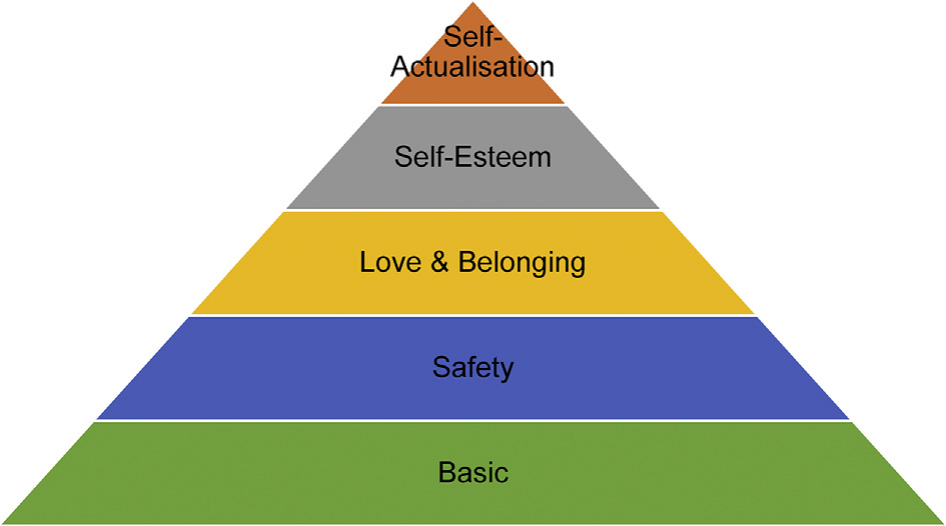
Maslow's hierarchy of needs.

This paper defines children's needs according to Maslow Hierarchy of needs which includes basic needs, safety needs, love and belonging needs and self-actualization needs. With the Child Welfare System primarily focusing on children's basic needs, it would seem to resemble the widely accepted Maslow Hierarchy of Needs (Maslow, 1943; 1954). Psychologist Maslow (1943) suggests that human beings are motivated by mainly five categories of needs. These needs are categorized as; basic; safety; love and belonging; self-esteem and self-actualization needs. Figure 1 illustrates the hierarchy of needs proposed by Maslow. The Maslow hierarchy of needs was used as a framework to analyze children's data.

### 1.2 Rationale & objectives

Though Botswana has invested in social protection, there are pockets of poverty persisting especially in rural and remote areas. With the dearth of literature on Botswana child welfare system as well as deficiencies in involving very young children as informants in popular discourse about their interventions, this paper sought to determine how children perceive themselves and their environment. The objective of the research was to determine the needs of general population of children. The study was carried out in Gaborone city-representing urban and Letlhakeng village-representing rural. This paper is based on my doctoral thesis (Ramabu, 2018), which sought to determine how Botswana's child protection system safeguards children from sexual abuse.

## 2 Theoretical framework

### 2.1 Systems theory

**Figure 2:**
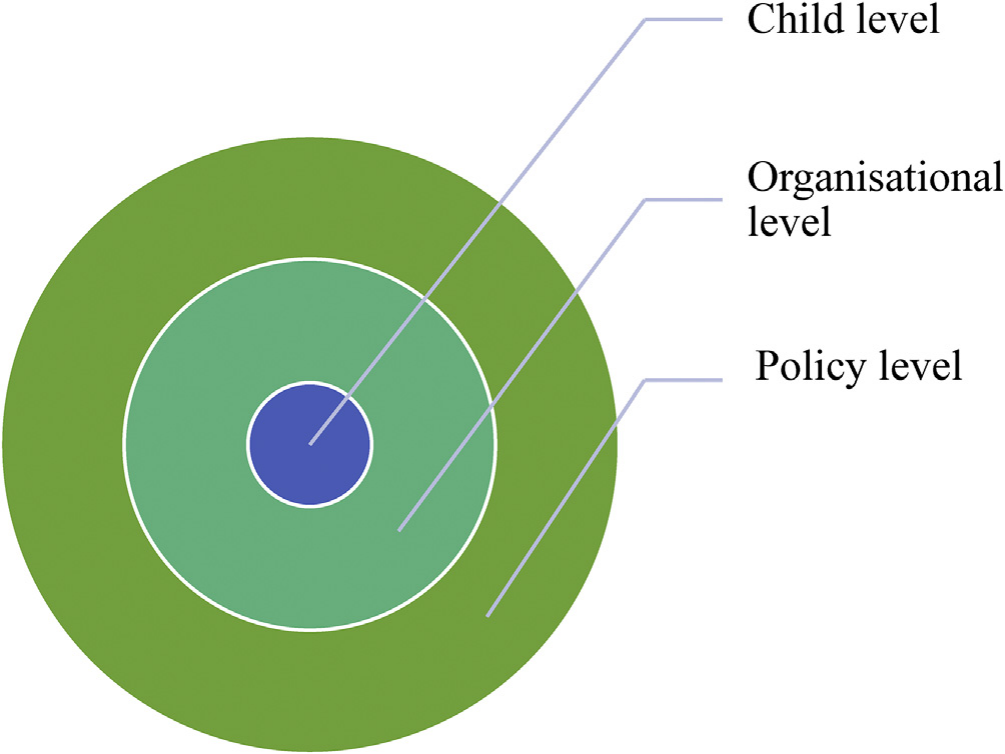
Child welfare system.

The systems theory as outlined by von Bertalanffy (1968) and recommended by United Nations (2006) as a suitable framework to comprehend child protection as well as a linking ideology across child protection agencies, underpins this study. This paper defines a system as Bertalanffy (1969: 30) who purports a system to be “*an organized whole made up of components that interact in a way distinct from their interaction with other entities and which endures over some period of time”* Therefore, the general systems theory is a general science of wholeness. In this paper, the term system is used to describe a set of child protection programs (UNICEF, 2008; Robalino et al., 2012) which are not limited to policy, human and financial capacities, stakeholders, children and communities, perpetrators, implementing systems structures and monitoring and evaluation systems. General systems theory is seen as a holistic framework within which to place child protection policy and practice. Botswana as an upper middle-income country (Botswana, 2016) has the capacity and resources to implement this systems vision. This paper focused on policy, organizational and child level as illustrated by Figure 2.

## 3 Methodology

This was a non-experimental study that collected qualitative data from primary data sources which included 1) draw and write technique [road of life and, a happy house and a sad house] with children aged 5–15 years; 2) in-depth interviews with program implementers at national-, district-, and service delivery-levels; 3) semi-structured interviews with caregivers and sixteen year-old Gaborone city children; 4) focus group discussions with sixteen year-old Letlhakeng village children. A total of 75 children, 26 key informants, 2 focus group discussions, 6 semi-structured interviews were conducted.

### 3.1 Analysis

Deductive approach was utilized to analyze qualitative data (in-depth, FGDs and semi-structured interviews) using the NVivo data analysis computer software (Bazeley et al., 2007). Children's drawings were given pseudo names, to aid with the presentation of the results. Some of the Setswana names given were Mpho, Kitso, Botho, Kutlo, Tshepo, Bonno and Lorato. Content analysis (Neuendorf, 2016; Krippendorff, 2012) was employed to analyze children's drawings. Maslow Hierarchy of Needs (1953) was used as a framework for the conversion of children's drawings to quantitative data. This provided a directed form of content analysis whereby, priori codes were derived from Maslow needs categories and then applied to the qualitative data. The main reason for quantitizing children's narratives was so that, I could extract meaning from qualitative data. The children's data was then analyzed using SPSS software version 22 (Pallant, 2013) for descriptive statistics. The graphs were generated using Excel and SPSS version 22.

### 3.2 Sampling

The sample size for child participants was predetermined and was fifty (twenty-five children per site). However, the sample size changed with circumstances on the field. For instance, Gaborone city children were interviewed at home, and that reduced the sample size. Nonetheless, interviewing Gaborone city children at home assisted in including children from independent schools who were initially not part of the study. Systematic sampling was employed. Two out of three schools were selected from Letlhakeng village. It is important to highlight that the sample size for Letlhakeng village children doubled due to use of FGD method among sixteen-year-olds, instead of planned semi-structured interviews. The in-depth interviews were purposively sampled to focus on participants that were involved in child protection system.

#### 3.2.1 Ethical consideration

Since this was a Doctor of Philosophy research study, ethical approval was obtained from Leeds Beckett University Institutional Review Board, research ethics application project no 304. The research was conducted in Botswana and therefore, obtained ethical approval from Human Research Documentation Committee (HRDC), the ethical clearance reference number PPME-13/18/1 vol VIII (470). For data to be collected from children in school, written guardian consent was obtained from the head teacher and guidance and counselling teacher. For those children interviewed at home, written consent was obtained from parents. Children aged 16 years were able to consent for themselves and. All children assented to participate in the study. They were two children in the whole study that did not assent to participate in the study and therefore, were excluded. For the data to be collected among village research participants, an assent was sought from the Kgosi before recruiting community members to participate in the study (Ramabu, 2019). Due to the sensitivity of the subject under study as well as the discomfort of the study participants to be audio-taped, only 3 in-depths interviewees and 2 focus group discussions consented to be audio-taped. As part of adhering to Botswana ethical consideration, the research findings were disseminated to Botswana community and policy makers.

## 4 Reflexive research

My perspective on this study was informed by my position as a black native from Botswana with South African parents (South African language and Botswana culture), having worked as a Child Nutrition Practitioner, a Nutrition Policy Maker, an Intercultural Researcher, a victim of domestic violence as well as being a survivor of Child Sexual Abuse. Additionally, I have studied and lived part of my adult life in England. That is, I carried out this research and interpreted findings through the lens of these multiple identities. It is against the backdrop of my historical, political and economic experiences that the study findings could be understood.

## 5 Findings

### 5.1 Participants characteristics

There were 53 children from Letlhakeng village who participated in the study. Of the 53 children from Letlhakeng village, 20 participated in two FGDs (10 children per FGD). All children from Letlhakeng children were in formal education and attending government schools. Their minimum age was five years, maximum age 16 years with mean age of 11.5 years (SD ± 3.1 years). Most of these children were aged 9–15 years. The most represented age group was 11–14 years. Of the 11-14-year-olds, 30.3% were still attending primary school. Age range of primary school children from Letlhakeng village was 5–16 years. There were slightly more girls than boys. Children who lived with both parents were almost the same number as those who lived with one parent. This one parent most children reported living with was the mother. Only 2 children reported living with grandparents.

Whereas, 22 children from Gaborone city participated in this study, with 3/22 children over 16 years participating in semi-structured interviews. Their minimum age was six years, with a maximum age of 16 years and mean age of 11.0 (SD ± 3.4). Slightly more children attended government schools, than children attending independent schools. The children living with both parents were in equal proportion to those who lived with one parent. Three children from Gaborone city reported living with other relatives such as uncle and aunt but not grandmothers.

When children's demographic characteristics from both sites were combined (n = 52), the boys and girls were in equal proportion. Most children (75%) were aged 9–15 years. Children living with both parents were in equal proportion to children living with one parent. Most children (86.5%) were in government schools. Table 1 depicts children's characteristics.

**Table 1:** Child participants characteristics (n = 52).

Characteristics	Gender		Age (Years)				Living Arrangements			School Type	
	Male	Female	5–8	9–15	16–18	Mean Age	Both Parents	One Parent	Other∗	Government	Independent
Gaborone city (n = 19)	11	8	4	13	2	11.0 (SD ± 3.4)	8	8	3	12	7
Letlhakeng village (n = 33)	15	18	5	26	3	11.5 (SD ± 3.1)	16	15	2	33	0
Total (n = 52)	26	26	9	39	5	11.3 (SD ± 3.1)	24	23	5	45	7
Total (%)	50%	50%	17.3%	75%	9.6%	_	46.2%	44.2%	9.6%	86.5%	13.5%

∗ Grandparents, uncles, aunties and other relatives.

### 5.2 Basic needs

Basic needs identified among children were food, shelter and clothing. Based on the data, only 3 of 19 children from Gaborone city expressed the need for food. Gaborone city children that expressed the need for food were in the 6–9 age category; whereas older children did not express the need for food. All children from Gaborone city did not express the need for clothes or shelter.

**Figure 3:**
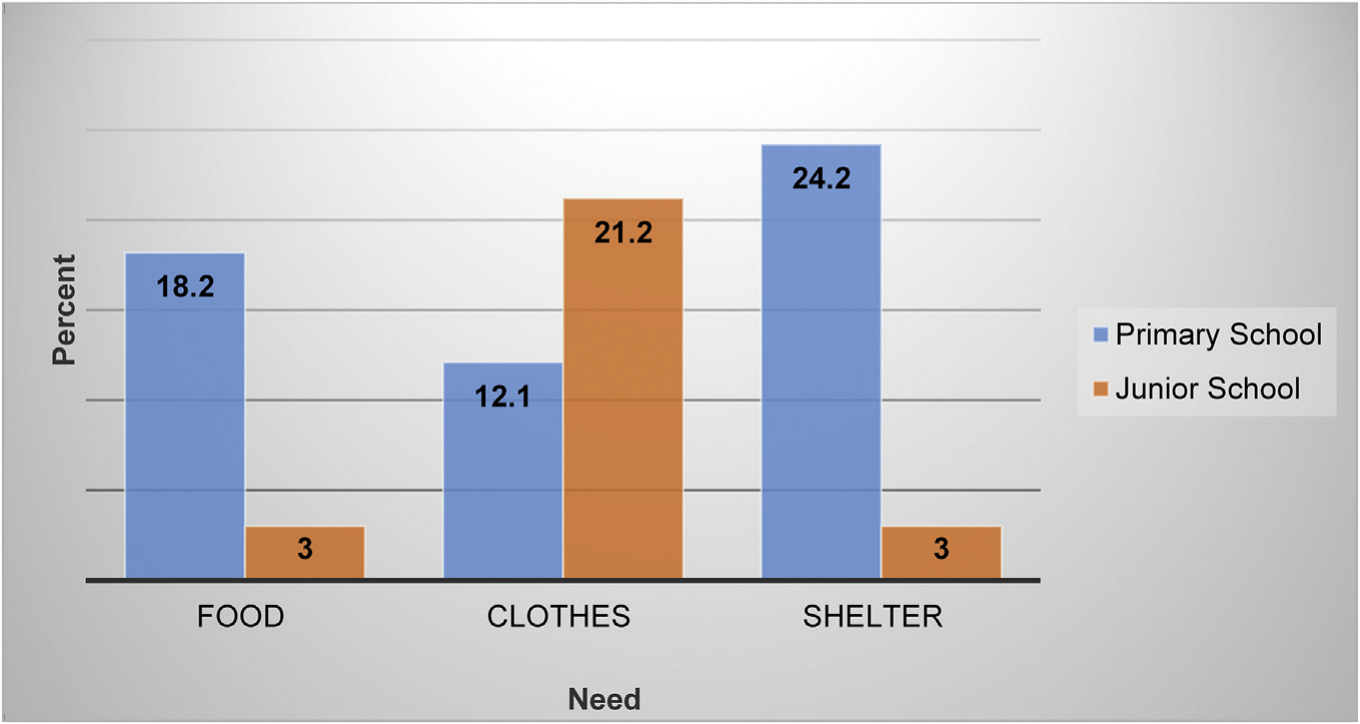
Letlhakeng village children's basic needs (n = 33).

On the other hand, primary school children from Letlhakeng village expressed the need for food compared to secondary school children. Letlhakeng village secondary school children were concerned with lack of or need for clothing compared to primary school children. Living in a comfortable and well-kept house was reported to be desired among Letlhakeng village primary school children but less of concern among secondary school children. Figure 3 depicts Letlhakeng village children's basic needs as categorized by school type.

Overall (n = 33), children from Letlhakeng village reported clothing to be what they would desire most (33.3%); followed by the shelter (27.3%); and then food (21.2%). Comparing children's basic needs between study sites, Letlhakeng village children were concerned with basic needs compared with Gaborone city children. Young children (5–9 years) from both sites expressed the need for basic needs compared to older children (9–16 years).

### 5.3 Safety needs

**Figure 4:**
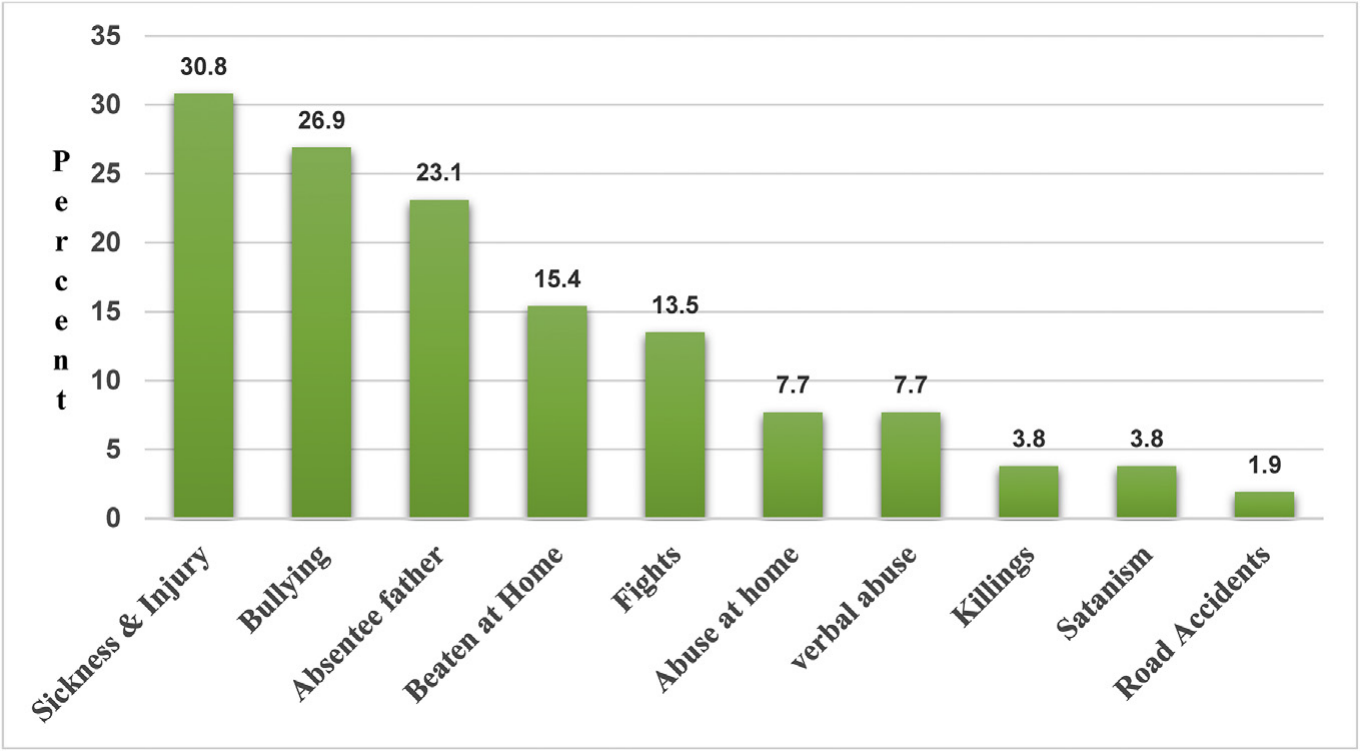
Combined safety needs (n = 52).

Safety concerns expressed by Letlhakeng village children such as absentee fathers, bullying, being beaten at home, sickness and injury were similar concerns among Gaborone city children. All children who reported being beaten at home indicated that the mothers were the ones who administered corporal punishment. When safety needs were categorized according to gender, slightly more boys (7/52) than girls (4/52) expressed the concern about the absence of a father. Furthermore, 2/52 from Gaborone city expressed a concern of an emotional absence of a father. When categorized according to age, bullying was slightly more of a concern among young children (9/52) than older children (5/52). Other safety concerns such as killings, Satanism and road accidents were a concern among a very small proportion of children. A small proportion of children reported to self-care during the day. However, a small proportion of children (3/52) reported feeling safe. Safety concerns of children from both locations are illustrated in Figure 4.

Children's narratives from FGDs and semi-structured interviews reflected safety concerns such as, sexual abuse and killings. These fears were succinctly described as;

*"We are afraid of being killed especially for rituals…we are afraid of being raped. We have heard stories of children as young as nine years raped in our village"–* 16-year-old girls' Focus Group Discussion, Letlhakeng village.

The Absentee father need though a love and belonging need, was considered a safety need as children reported it as a response to the question; *what makes you not feel safe?* The following are some examples of children's narratives about their fathers that have been quantified and presented in Figure 4.

A fifteen-year-old girl from Letlhakeng village illustrated with a smiley face on her road of life drawing that, she visited her father for the first time when she was fourteen years old.

A six-year-old boy from Gaborone city indicated that a happy house is the one *“without my father”.*

A fifteen-year-old boy from Letlhakeng village indicated with a sad face on his road of life that his father “*denied me*" when he was one-year-old. This implies that the father denied paternity and failed to take responsibility for the child. The child further expressed that he would feel safe if he and his father could visit each other.

“*I live with both of my parents. But my father and I do not interact. I cannot talk to him and he doesn't talk to me. I was fortunate that when I reached adolescence a male teacher at school took interest in me. I could have become anything*”-16-year-old boy, Gaborone city

Additionally, Respondents' narratives reflect the involvement of a father in children's lives to be a challenge. As some Respondents indicated;

*The children have their mothers. The fathers do not guide boy children. "Banna ga ba yo" loosely translated to imply that men are absent [emotionally, physically, economically]. They are absent while present. They watch television, eat, read a newspaper and then go to bed* - UNICEF, Botswana.

*Children are left to be given parental guidance by the television. The television is the best mother, and it is the best father. We don't even know what they watch. It's them who know. Even if I can be home and they [children] want to interact with me, I will say "le a nthodia" loosely translated to imply that’‘you are making nose and disturbing me’‘. So, our children are learning more things through technology than us. How we balance this I don't know; I don't have the answer. It has a lot of implications for child sexual abuse. Whether the father is married or not, the child needs to know the parent. If you are the father, take responsibility*–Gender Officer, Civil Society.

*Children do not have guidance. The issue of bullying. I grew up my father telling me that a woman is not supposed to be beaten. My father will even ask me if I have ever seen him beat my mother. Like I was saying our kids are guiding themselves. The kids get raped when they idle in shopping complexes. I heard of a child who got raped in the toilet because the child went to the toilets alone without parents.* OVC Technical Officer, Civil Society.

Furthermore, the law was reported to compound this absentee father challenge experienced by children. As one Respondent indicated;

*Botswana laws compound the problem of absentee fathers by giving child custody automatically to women even if they [women] could be irresponsible. Therefore, men are left out from the start especially in situations where they [parents] were never married.* –Primary School Guidance and Counselling Teacher, Letlhakeng Village.

### 5.4 Love and belonging needs

**Figure 5:**
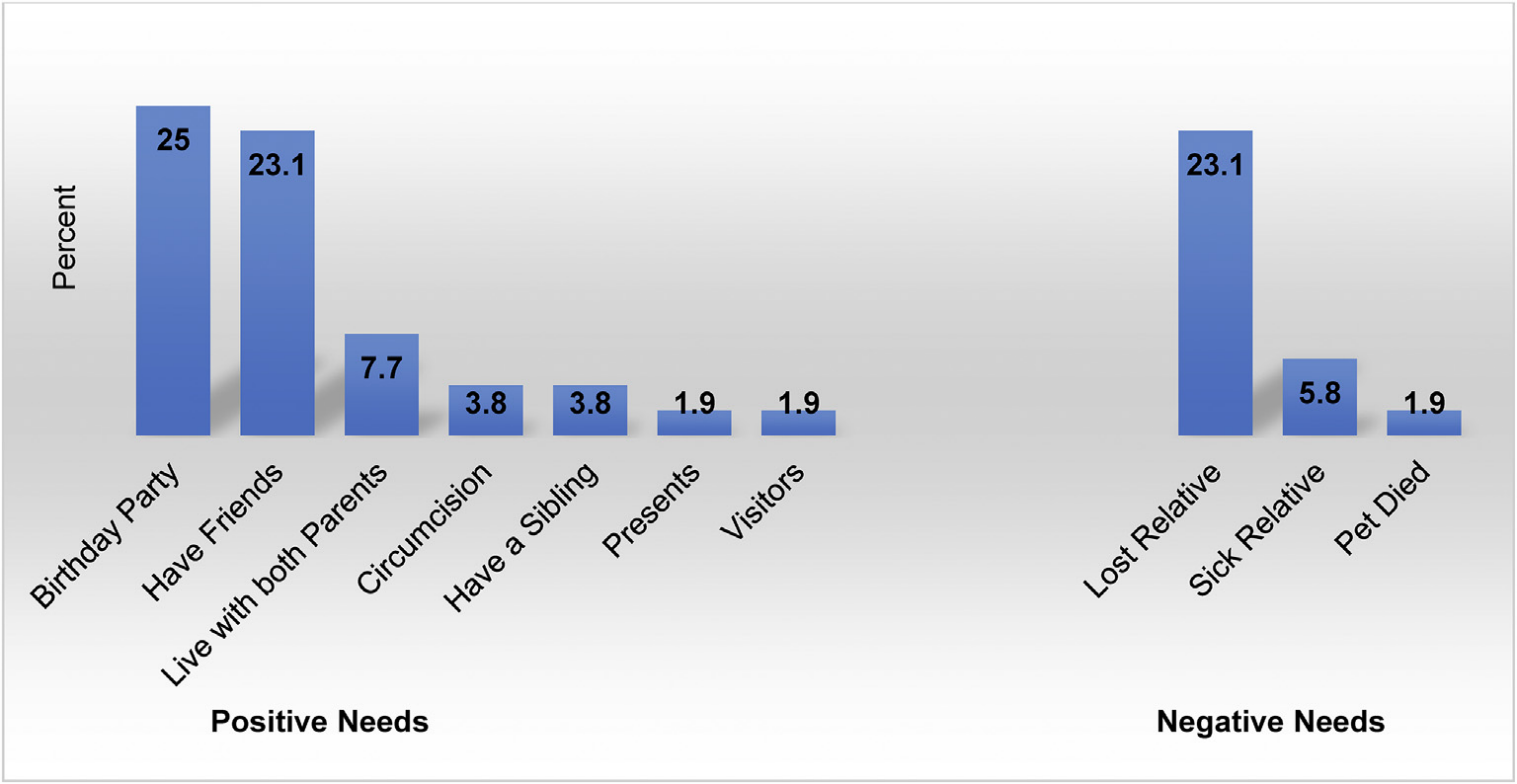
Combined love and belonging needs (n = 52).

Children's combined love and belonging needs show that, having a birthday party and having friends were children's main positive love and belonging needs. Whereas, losing a relative was children's major negative love and belonging need. A small proportion of children reported a sick relative as a negative love and belonging need. Whereas a small proportion of children expressed feeling happy when living with both parents. Figure 5 illustrates children's combined needs.

Respondent's narratives reported addressing psychosocial needs of OVC children as indicated in the response below

*“…have a psychosocial support that we take children to retreat camps. It is a grief and bereavement program for children who have lost relatives. You will find out that some of these issues of sexual abuse come out. Children end up talking about the sexual abuse that they never reported”-* Social services, Gaborone city

### 5.5 Self-actualization needs

Children in this study mainly reported school performance (13/52) which is considered a self-actualization need. Of the 13/52 children who reported to be happy with school performance, older children (11/52) were more represented than younger children (2/52). Whereas one child reported wanting to become a doctor, and another child reported to be in fulfilment of his dream of becoming a pop artist.

**Figure 6:**
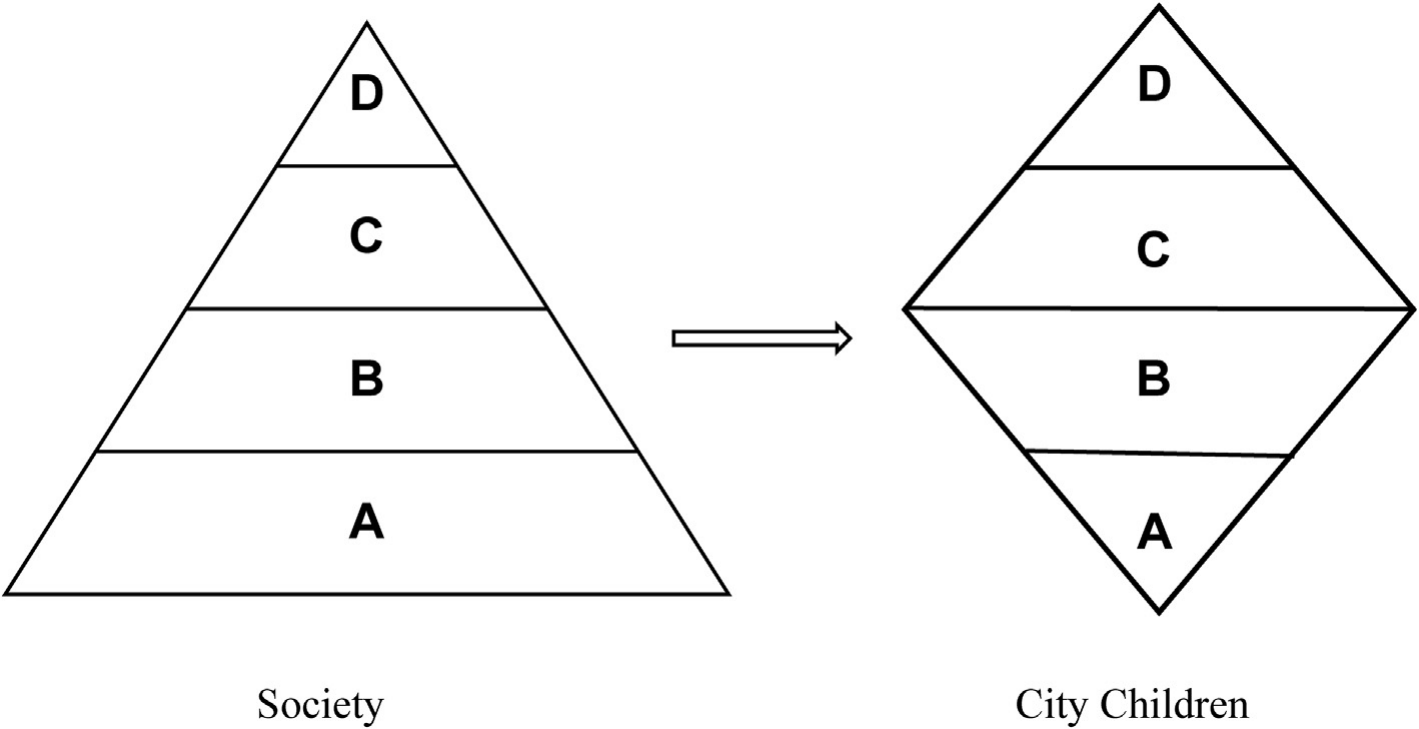
Hierarchy of needs changes in the city.A represents the basic needsB represents the safety needsC represents love and Belonging needsD represents the self-actualization needs

Overall, children's location seems to configure the Hierarchy of Needs. Children from Letlhakeng village needs depicted the proposed Maslow Hierarchy of Needs. However, though Gaborone city children sample size was small (n = 19), the city circumstances reflect to be a different situation from the whole society. The changes are shown in Figure 6.

**Figure 7:**
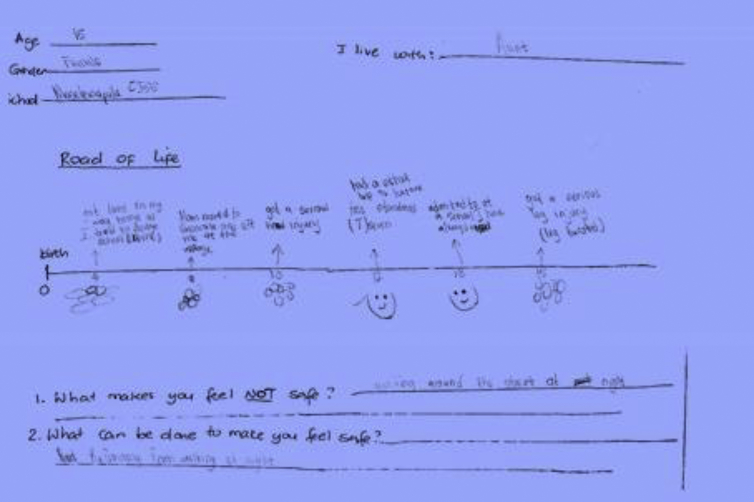
Lorato's road of life.

Source: Lorato's Drawing, girl, 15 years, Gaborone City (18/12/2014).

Some of the children's drawings are included here for the reader to appreciate how children expressed themselves (see Figure 7).

**Figure 8:**
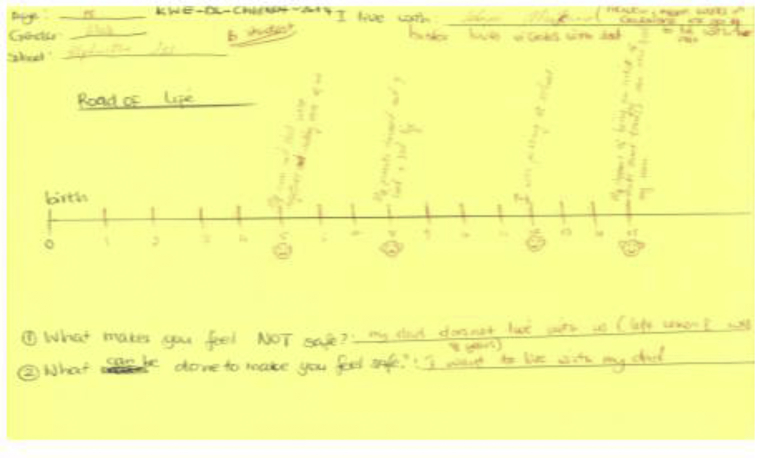
Mpho's road of life.

Source: Mpho, 15 years, Letlhakeng Junior Secondary School (26/11/2014).

In her picture, Lorato talks about school and injuries sustained. She talks about; trying to dodge school when she was four years old and getting lost; having a school safari trip to Kasane [Chobe], and being admitted to the school she likes. Lorato's extracts provide details about the two serious injuries she sustained in which she twisted her leg. She also mentions her concern of living outside parental care by indicating that *"mom moved to Gaborone city and left me in the village".* Lorato lives with her aunt. Overall, Lorato mentioning living outside the care of her mother, suggests that she belongs with her mother (see Figure 8).

**Figure 9:**
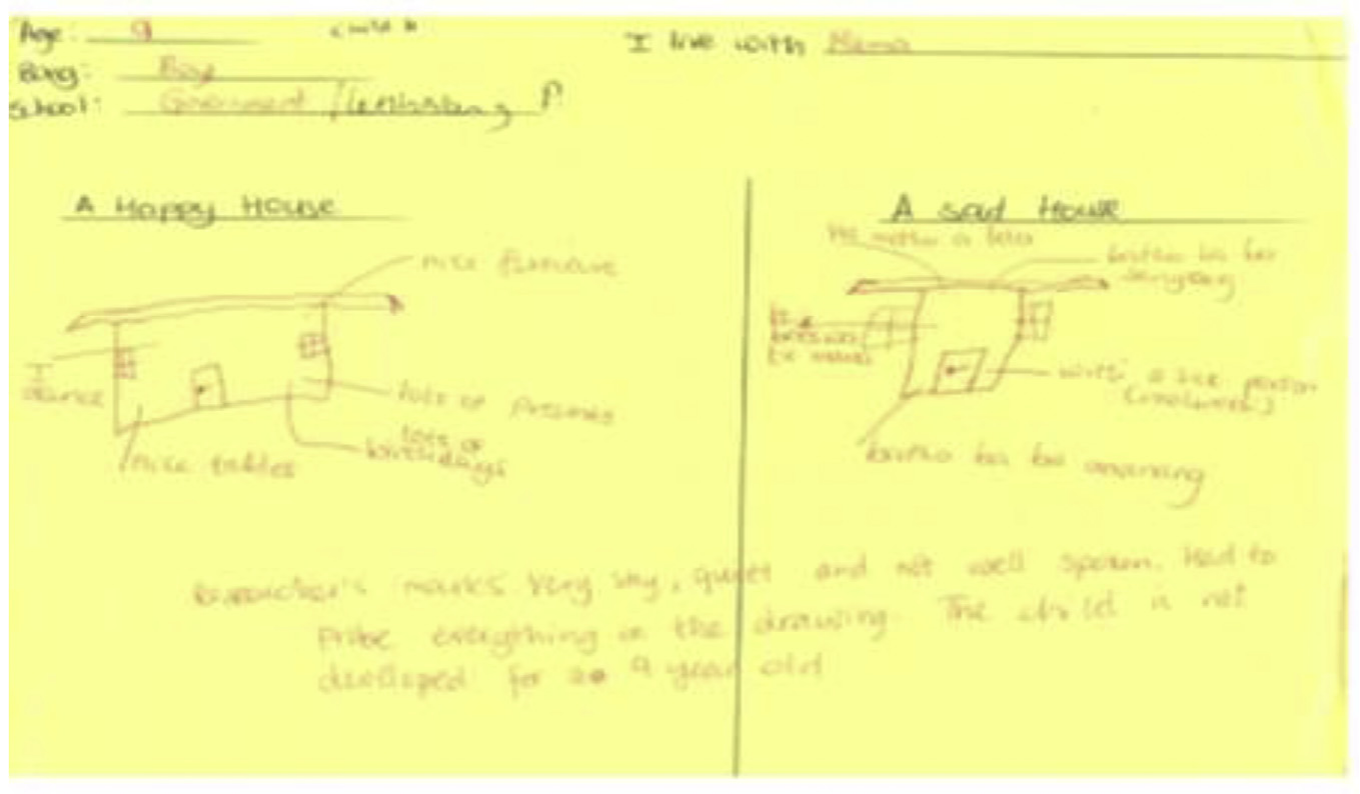
Botho's happy and sad house.

Source: Botho's drawing, nine years, Letlhakeng village (19/11/2014).

Mpho who lives with the grandmother in Letlhakeng village writes on his drawing that, his sister lives with his father in Gaborone city. His mother also works in Gaborone city. In this extract, Mpho mentions that he used to live with his parents and he was very happy. Mpho narrates that by the time he was eight years old, "*my parents divorced and I lived a sad life”*. The separation of Mpho's parents could be contributory to him living with his grandmother in Letlhakeng village, while his parents live but separately in Gaborone city where they both work. However, Mpho's school performance seems not affected, and he is happy that he is still doing well in school. Mpho also reports realising his dream of becoming a pop artist which makes him happy because *“he can now feed his mum”*. Mpho reports that he is going to live with his mother in the future. By Mpho mentioning residing with his mother illustrates that he sees his living arrangement temporary and his home is with his mother. With Mpho's concern about living outside parental care, it is not surprising that his safety concern is that his father does not live with him and he would feel safe when living with his father. This overall description highlights Mpho constructing his living arrangement in relation to a two-parent household (see Figure 9).

In this scenario, Botho who lives with his mother only pictures a happy house where he has a lot of birthday parties, a lot of presents and dances a lot. This is the house where Botho imagines it would have "*nice furniture*” and ‘*nice tables*'. Whereas, a sad house appears smaller than the happy house. Botho associates a sad house with emotional and physical abuse. Botho narrates that, a sad house is where; his mother beats him; where somebody cries; there is a sick person; and where people argue. Though I indicated on Botho's drawing that *“he is very shy, quiet and not well spoken and I had to probe everything on the drawing”*, he seemed to paint a clear picture of the two houses and managed to express his emotions.

## 6 Discussion

### 6.1 Basic needs

Primary school children's narratives reflecting the need for food compared to secondary school children is in concordance with literature in Botswana (Nnyepi et al., 2011) where, younger children have been shown to be disproportionately affected by malnutrition than older children. The village children showing a need for basic needs compared to city children resonates with empirical data (World Bank, 2013), which posits poverty to be higher in rural areas in families raising children. However, most of the empirical data available on basic needs and female-headed households are from rural areas and less from urban areas. According to Acquah et al. (2014), the poorer Gaborone city has a large proportion of female-headed households and therefore, experience food insecurity similar to rural Botswana.

This insufficient basic needs reported by children in this study has multiple realities. One of the realities discovered in Botswana and other settings is that children could engage in risky sexual behaviours in order to survive (Mathoma et al., 2006; Anderson et al., 2012; SADC, 2011, Underwood et al., 2011; Tsai et al., 2011; Mustaine et al., 2014; Dahl, 2015). Therefore, the need for basic needs reported by village children in this study could heighten their vulnerability to sexual abuse. Sadly, this transactional sex occurs against the backdrop of HIV/AIDS, which could increase the risk of children acquiring the HIV.

### 6.2 Safety needs

With regards to safety needs, the use of corporal punishment reported to be administered by mothers is borne out by evidence of studies elsewhere (Breen et al., 2015) including the work of Osborne et al. (2016) which discovered parenting to be a gendered exercise where mothers spend more time with children. This safety concern challenges the UNCRC rights based assumption that all children should be afforded protection from all forms of abuse. However, challenges remain in violence prevention interventions in the middle-income context such as Botswana.

Physical bullying which was reported by a significant number of children in this study is in congruent with Shehu (2009) who posits physical bullying to be occurring in Botswana schools in the context of physical education and perpetrated by children of both genders. Empirical data on peer bullying has been mainly from adolescents (Fleming & Jacobsen, 2009). The evidence of this paper, however, suggests younger children in lower primary school experiencing bullying more than older children in secondary schools. This finding of younger children experiencing bullying than older children resonates with Rech et al. (2013) who purport younger children to be victims of bullying by older children, especially during physical education activities. Additionally, interest in bullying in Botswana schools is recent and need more research to explore. This paper extends knowledge on bullying and helps give visibility of the problem in Botswana and therefore, has implications for child health and well-being.

The presence of a father in a household was reported not beneficial by a small proportion of boy children in Gaborone city. Therefore, a father's lack of interaction with children though living in the same household, could leave children's emotional need for a father unrealised. This challenge of absentee fathers is not recent and has been appreciated in previous studies in Botswana (Schapera, 1941; Alverson, 1978; Jorosi-Tshiamo, 2013; Padi et al., 2014) as mainly perpetrated by labor migration. This absence of a father has been continued and sustained through generations as evidenced in this study. However, absentee father challenge is a complex and multidimensional phenomenon.

A small proportion of children from both sites reported living with grandparents and other relatives. This resonates with literature in Botswana where single mothers who work in urban areas send their children to live with their grandparents at the villages (Makwinja-Morara 2009; Van Allen, 2015). Makwinja-Morara (2009) argues that, cultural practice widely accepted of grandparents rearing children could encourage unplanned pregnancies and therefore could heighten the absentee father phenomenon. Whereas children from the city who lived with other relatives could be that rural parents send their children to the city to live with other relatives as a way of improving their children's standard of living.

Another safety concern reported by some children was being left to self-care. This challenge, however, has been documented in other studies in Botswana (Ruiz-Casaeres & Heymann, 2009) that found out that parents consider childcare choices and desire quality care for their children, yet their choices are limited. This is the situation that could be said to be societal rather that parental neglect. Ruiz-Casares & Heymann (2009) expound upon this point by highlighting a need for a full understanding of political, social and economic factors to better contextualise why parents leave children to self-care.

Given the analysis above, some of these safety concerns could render children vulnerable to sexual abuse. For instance, one Respondent's narrative reflects a child left to self-care and then, sexually victimised.

**Case study**

I had a 7-year-old child whose mother was unemployed. Due to lack of basic needs home, someone else accommodated the child. The child was unattended most of the time and ended up getting sexually abused. The people who were abusing her gave her sweets prying on her lack of food. The child (who was a girl) was abused by people she knew and lived on the same street with.- *Social Services, Gaborone city.*

### 6.3 Love and belonging needs

The findings from this study show children expressing happiness when having a best friend and sadness when deprived of a sense of belonging. Other children reported happy living with both parents and sad not living with parent(s). This social motivation has been reported elsewhere (Over, 2016) that claims that, humans are deeply dependent on relationships especially positive interactions. The children expressing the satisfaction of having a best friend or close friends is consistent with literature that support that individual's need to belong must consists of positive interactions within a framework of long-lasting affective concern for each other's welfare (Bausmeister & Leary, 1995).

Further, failure to support children during loss and grief, could also be located within socio-cultural perspective regarding the socialization of children which excludes children from family matters (Maundeni, 2002). On the other hand, Botswana culture would seem to have mechanisms to support children through loss and grief process. One way of communicating the loss of a loved one to a child reported by Sabone (2009) is whispering to a child while sleeping to protect them from trauma they could experience if they were told while awake. However, lack of involvement of children in grief and loss could be a result of caregivers who lack age-appropriate information on coping and loss, compounded by lack of grief and loss health promotion programs targeting the community.

### 6.4 Self-actualization needs

Majority of children who reported to be happy with school performance were 13 years and upwards. There is dearth of literature in Botswana in this area, with some recent studies focusing on children's image in light of obesity and feeding choices (Maruapula et al., 2011; Wrotniak et al., 2012; Malete et al., 2013). Further studies would be required to explore this highest need on the Maslow Hierarchy and its interaction with other level needs.

### 6.5 Systems approach to children's needs

**Figure 10:**
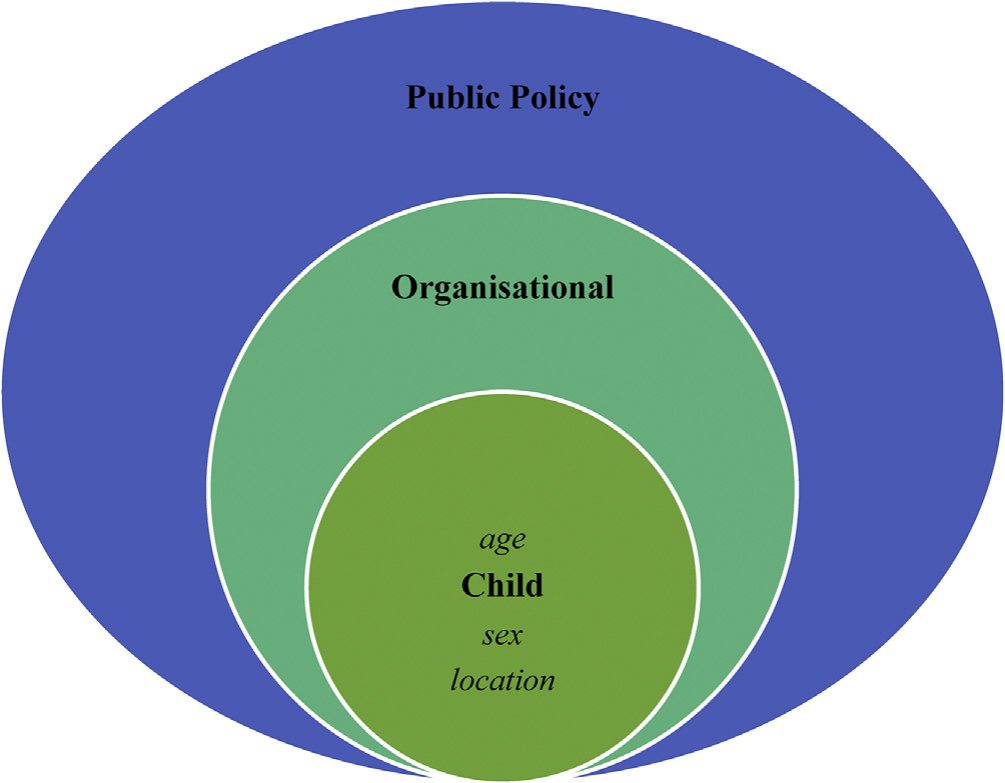
Systems approach to children's needs.

Children's needs could further be understood through a systematic lens. Figure 10 illustrate a systematic approach to children's needs.

At the interpersonal level, children have expressed happiness with having friends and living with parents. This sense of belonging and the need to live with both parents are well documented in the literature as a natural process of human interaction which could contribute positively to children's health and well-being (Bausmeister & Leary, 1995; Over, 2016). At the organizational level, children have expressed concerns with verbal and physical abuse they experience at school and home. Organizations, however, could be better located within the public and private sphere. Community-level factors could be better viewed regarding norms and practices surrounding child rearing which could cause children to live with unmet needs. These community-level factors such as construct of man as a breadwinner, have dictated that children mainly from the village grow up without the presence of a father. Furthermore, broader societal factors as provided for or not provided for in policies and laws, could be seen as creating an environment in which children's needs are overlooked and suppressed, a concept popularly referred to as seen but not heard (Lansdown, 2001; Maundeni, 2002). Essentially this paper promotes an understanding of the connection of children's lived experiences with other levels of the child welfare system.

### 6.6 Study limitations

This study should be considered alongside these limitations. The study excluded out of school children, and the sample size relatively small and therefore not generalizable to the general population of children. Generating data from children is still an emerging field with inherent difficulties. Thus, children's drawing has been shown to have limitation and should not solely be relied upon as the sole source of data (Katz & Hanama, 2013). This is mainly because the quality and reliability of children's testimonies could not be assessed in this study. Hence, data were solicited from different child protection stakeholders to augment limitations from children's data. However, drawing as a data collection tool has been studied and found to provide rich narratives from children without compromising the accuracy (Katz et al., 2014).

## 7 Conclusion

This paper has demonstrated that though Botswana is considered a high middle-income country, meeting children's needs remains a challenge far from being resolved. The children in this study's concern about their needs is consistent with those reported among OVC children in Botswana and elsewhere (Roets & Chakalane-Mpeli, 2007; Ministry of Local Government, 2008a, Ministry of Local Government, 2008b). Therefore, this paper advances a growing appreciation of the need to recognize that, all children are vulnerable by virtue of age and dependency on adults (Munro, 2011). Thus, childhood category remains a vulnerable age group which could have implications for their health and wellbeing. A greater concern with these findings is that this study was conducted among the general population of children who were not categorized as having vulnerability. For the general population of children's needs to continue unmet in an upper middle-income country with almost half of the population being children under 18 years (Jones & grant, 2009; Botswana Population census, 2011), is a concern needing further studies to comprehend.

## Declarations

### Author contribution statement

N. M. Ramabu: Conceived and designed the experiments; Performed the experiments; Analyzed and interpreted the data; Contributed reagents, materials, analysis tools or data; Wrote the paper.

### Funding statement

This research did not receive any specific grant from funding agencies in the public, commercial, or not-for-profit sectors.

### Competing interest statement

The authors declare no conflict of interest.

### Additional information

No additional information is available for this paper.
